# Dead space fraction indicates the titration of optimal positive end-expiratory pressure after recruitment in acute respiratory distress syndrome

**DOI:** 10.1186/cc9623

**Published:** 2011-03-11

**Authors:** Y Yang, J Chen, H Qiu

**Affiliations:** 1 Zhong-Da Hospital and College of Southeast University, Nanjing, China

## Introduction

The objective of this study is to evaluate the value of dead space fraction (VD/VT) as a method for indicating the optimal PEEP titration.

## Methods

Twenty-three patients with ARDS were enrolled in the study. After lung recruitment using sustained inflation (SI), the optimal PEEP was respectively titrated by the optimal oxygenation, the maximum static pulmonary compliance (Cst), and the lowest VD/VT. The influence of these methods on oxygenation, Cst, VD/VT and FRC were observed.

## Results

The PEEP level titrated by the lowest VD/VT (10.1 ± 2.8 cmH_2_O) had no significant difference from the PEEP level titrated by the maximum Cst (11.3 ± 2.5 cmH_2_O) (*P *> 0.05). However, the PEEP level titrated by the lowest VD/VT was significantly lower than that determined by optimal oxygenation (*P *< 0.05). The oxygenation at the PEEP titrated by VD/VT was significantly lower than the optimal oxygenation, but no significant difference in PaO_2_/FiO_2 _was observed between the PEEP titrated by the lowest VD/VT and the maximum Cst. Additionally, the VD/VT and the FRC at the PEEP chosen by the three methods also had no significant difference.

## Conclusions

The lowest VD/VT could be one of the methods to choose the optimal PEEP in ARDS patients.
